# Imaging manifestations of cervical aggressive fibromatosis: a case report and literature review

**DOI:** 10.3389/fonc.2024.1458486

**Published:** 2024-10-11

**Authors:** Zhilan Huang, Jinghong Li, Houyun Xu, Jiaying Liu, Tian Yang, Caijuan Zhang, Xuan Jin, Jibo Hu, Jun Yang

**Affiliations:** ^1^ Department of Radiology, The Fourth Affiliated Hospital of School of Medicine, and International School of Medicine, International Institutes of Medicine, Zhejiang University, Yiwu, China; ^2^ Department of Emergency Medicine, The Fourth Affiliated Hospital of School of Medicine, and International School of Medicine, International Institutes of Medicine, Zhejiang University, Yiwu, China

**Keywords:** aggressive fibromatosis, cervical, neck, imaging manifestations, low signal band

## Abstract

Cervical aggressive fibromatosis is a rare intermediate tumor characterized by invasive growth. Aggressive fibromatosis (AF), also referred to as desmoid-type fibrosarcoma or grade I fibrosarcoma, is a clonal proliferative lesion of fibroblasts located in deep soft tissues. While many cases have been reported, there are relatively few involving aggressive fibromatosis in the anterior inferior margin muscle space of the neck trapezius muscle. We present a case of pathologically confirmed left cervical aggressive fibromatosis, admitted to the Fourth Affiliated Hospital of Zhejiang University. The initial ultrasound and magnetic resonance imaging (MRI) scan of the patient revealed a mass in the left cervical dorsal muscle space, which slowly increased after one year. An enhanced MRI scan initially diagnosed the mass as a left cervical schwannoma. The patient underwent neck soft tissue lesion resection surgery, with postoperative confirmation of cervical aggressive fibromatosis. Our case suggests that fibromatosis cannot be ruled out, and the low-signal cord-like non-enhanced areas, representing collagen fiber characteristics after enhanced scanning, are significant imaging features in diagnosing cervical fibromatosis. Based on the available literature, we have conducted preliminary research on the clinical presentation, imaging manifestations, diagnosis, and differential diagnosis of cervical aggressive fibromatosis to improve clinical understanding and ensure timely clinical treatment.

## Introduction

AF was first described by Muller in 1838 (1). Fibromatosis is a rare fibroblastic lesion that infrequently occurs in the head and neck (2, 3), though it can present with malignant characteristics of invasive growth. Head and neck invasive fibromatosis accounts for less than 6%-8% of systemic invasive fibromatosis cases and 7%-25% of all extra-abdominal cases. The complex anatomical structure and critical location of this region pose significant risks to an individual’s quality of life and safety. Few cases in the published literature describe invasive fibromatosis originating from the anterior inferior margin of the trapezius muscle space in the neck. Reports indicate that the recurrence rate of aggressive fibromatosis in the neck is higher than that of non-head and neck fibromatosis (4). In clinical practice, the diagnosis and management of aggressive fibromatosis of the neck present considerable challenges. The invasive nature of cervical aggressive fibromatosis complicates both diagnosis and treatment, as it not only grows rapidly but also extensively infiltrates surrounding tissues, disrupting the normal function of adjacent structures. Patients may exhibit non-specific symptoms in the early stages, such as neck lumps and pain, which are often mistaken for more common neck conditions. Due to its rarity, clinicians may have limited familiarity with this condition, increasing the risk of misdiagnosis or missed diagnosis. In terms of treatment, the invasiveness and location-specific nature of cervical aggressive fibromatosis make treatment strategies particularly challenging. Surgical resection is generally the preferred approach, but completely removing the tumor while protecting critical surrounding structures (e.g., blood vessels, nerves) remains an urgent problem. Here, we report a case of cervical aggressive fibromatosis and describe its dynamic imaging findings over two years to improve clinical understanding of this condition.

## Case presentation

The patient, a 36-year-old female, reported noticing a mass in her left neck one year prior to admission, which slowly enlarged gradually increased in size and was accompanied by mild tenderness. The texture and flexibility of the mass remained acceptable. The patient told her doctor that she had received local massage treatment in an external hospital. The patient had no prior history of trauma, tumors, or familial diseases related to the head and neck region. Upon general physical examination, the patient’s body temperature was 36.8°C, pulse rate was 69 beats per minute, respiratory rate was 18 breaths per minute, and blood pressure was 102/63 mmHg. Her height was 171 cm, weight was 61.7 kg, resulting in a Body Mass Index (BMI) of 21.1 kg/m^2^. A tumor approximately 5×6 cm in size was palpable in the left neck region, with slight pain. The mass remained flexible, and there were no signs of tension or involvement of the nervous system (NS). Laboratory analysis showed a reduction in the percentage and absolute count of eosinophils, while coagulation function, as well as liver and kidney function tests, were all within normal limits. Additionally, tumor markers, including carcinoembryonic antigen (CEA), glycan antigen, cytokeratin 19 fragment, alpha-fetoprotein (AFP), and squamous cell carcinoma-associated antigen, were all within normal ranges.

**Figure 1:**
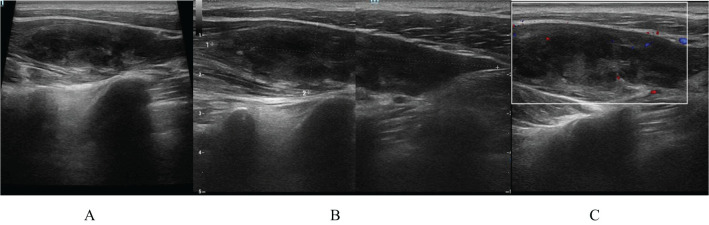
Ultrasound examination on April 17, 2022: **(A, B)** A hypoechoic mass measuring approximately 1.76×7.64 cm is observed in the lower trapezius muscle of the left neck. The mass has a clear boundary, a regular shape, and a spindle-like appearance. **(C)** The lesion exhibits a small amount of punctate blood flow signal.

On April 17, 2022, an ultrasound examination revealed a hypoechoic mass measuring approximately 1.76×7.64 cm in the lower portion of the trapezius muscle in the left neck (**Figures 1A, B**). The mass had a clear boundary, regular shape, and spindle-like appearance. A small amount of punctate blood flow signals was also observed within the lesion (**Figure 1C**), suggesting a low-echoic mass in the soft tissue of the lower left trapezius muscle. Schwannoma was considered a possible diagnosis.

**Figure 2:**
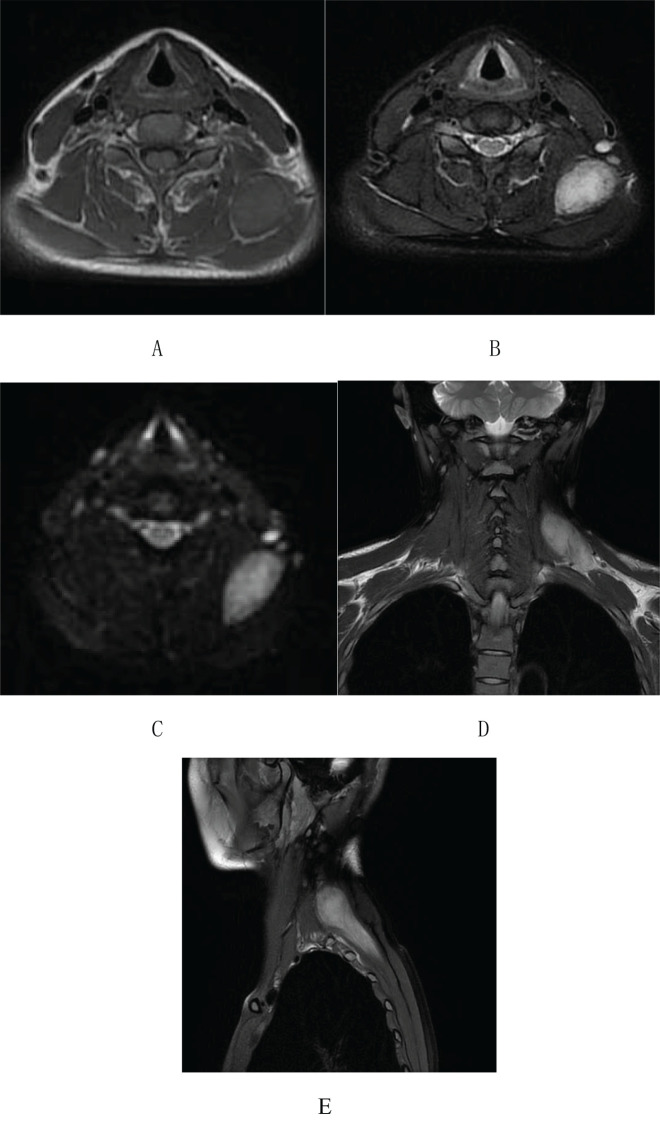
Neck soft tissue MRI plain scan on April 18, 2022: **(A)** An abnormal mixed-signal lesion, approximately 28×45×66 mm in size, is visible in the space between the dorsal muscles of the left neck. T1-weighted imaging shows an equal signal with low-signal stripe shadows within the lesion. **(B-E)** T2-weighted imaging and DWI reveal a mixed high signal with distinct boundaries and low-signal stripe shadows internally.

**Figure 3:**
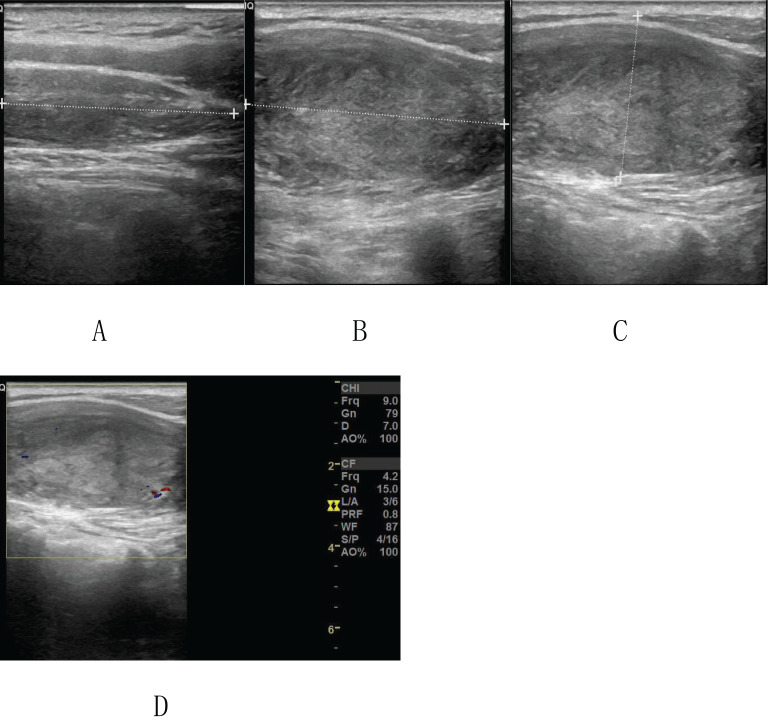
Ultrasound image on August 14, 2023: **(A-C)** A hypoechoic mass measuring approximately 2.82×8.71 cm is visible in the lower trapezius muscle of the left neck. The mass has a clear boundary, regular shape, and a spindle-like appearance. **(D)** A small amount of punctate blood flow signal is observed within the lesion.

On April 18, 2022, the patient underwent a Magnetic Resonance Imaging (MRI) plain scan of the soft tissues in the neck. The MRI revealed a mass-like lesion with mixed signals with a clear border in the dorsal muscular gap of the left neck, and low-signal strand-like structures were visible within it (**Figures 2A-E**). Additionally, multiple slightly enlarged lymph nodes were observed on both sides of the neck. As the MRI scan was inconclusive regarding the nature of the lesion, the clinician recommended an enhanced scan for further evaluation. By August 14, 2023, the patient noticed that the mass in her left neck had gradually increased in size over the past year. She decided to seek further hospitalization for diagnosis and treatment. Another superficial mass ultrasound was performed, which showed that the lesion (**Figure 3**) had enlarged compared to the ultrasound findings from April 17, 2022.

**Figure 4:**
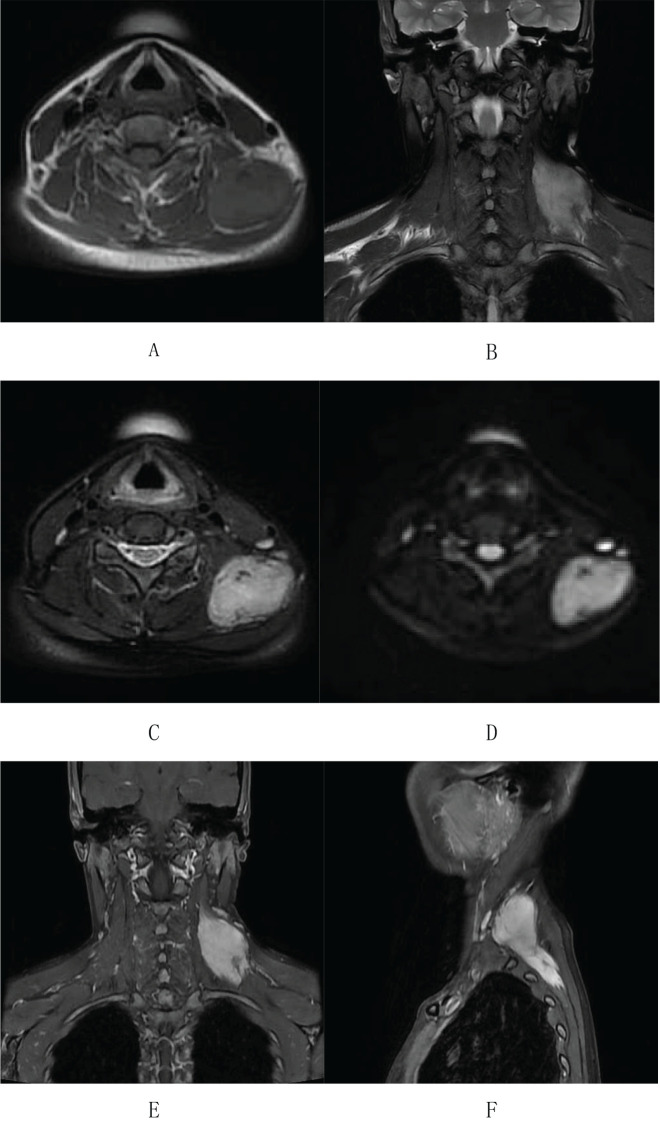
Enhanced MR imaging of neck soft tissue on August 15, 2023: **(A-C)** An abnormal signal lesion, clump-shaped, is located in the anterior inner border muscle space of the left dorsal trapezius muscle. It shows equal signal intensity on T1-weighted imaging. **(B-D)** T2WI and DWI display a mixed high signal with clear boundaries. The lesion measures approximately 29×50×81 mm. **(E, F)** After enhancement, the lesion shows significant enhancement with a few low-signal cord-like shadows visible within.

On August 15, 2023, the patient underwent an enhanced MRI scan of the soft tissues in the neck. The results revealed that the mass in the muscular gap beneath the anterior trapezius muscle on the back of the left neck had enlarged compared to the MRI findings from April 17, 2022. After enhancement, significant intensification was observed (**Figures 4E, F**). A few low-signal strand-like structures were visible within the lesion (**Figures 4A–F**). Multiple slightly enlarged lymph nodes were noted on both sides of the neck, some of which had increased in size since the previous examination. The clinician thought that this feature might be consistent with schwannoma and could not exclude fibromatosis.

After doctors thoroughly discussed the patient’s condition with her, the patient actively participated in the treatment. On August 16, 2023, the patient underwent surgery for left neck tumor resection. The anesthesia method used was a combination of intravenous and inhalation general anesthesia. Surgical procedure: The patient was placed in a supine position, and an incision was made over the tumor, following the direction of the skin lines. The skin and subcutaneous tissue were incised to expose the trapezius muscle, which was then separated. During surgery, the tumor was found beneath the trapezius muscle, with a hard texture and a relatively clear boundary. The surgeons carefully freed the tumor from the surrounding tissues and severed the muscle fibers connected to it. Although the boundary of some regions of the tumor was not entirely clear, the tumor was completely resected under visual inspection. The surgical area was repeatedly rinsed with hydrogen peroxide and iodine physiological saline. Hemostasis was achieved, and a subcutaneous drainage tube was inserted, connected to negative pressure. The muscles and skin were then sutured layer by layer. The patient experienced no complications during the surgery, and the intraoperative blood loss was approximately 20 ml. Postoperatively, the patient received an intravenous infusion of 1.5 g cefuroxime sodium every 8 hours for 24 hours to prevent infection. The incision dressings were regularly changed by the medical team. The subcutaneous drainage tube drained a total of 88 ml of pale blood-tinged fluid 13 hours after surgery and 34 ml of pale blood-tinged fluid 24 hours after surgery. The drainage tube was removed 48 hours post-surgery, and postoperative high-pressure injury protection care was provided for 8 hours. Symptomatic treatment included the intravenous infusion of 50 mg flurbiprofen axetil every 12 hours for 72 hours to manage pain. The surgical specimen was sent for routine pathological examination.

**Figure 5:**
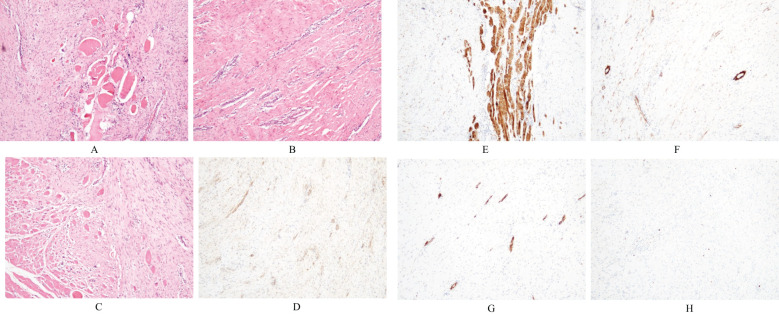
Histopathological images. **(A)** Histopathology from the left neck shows spindle-shaped tumor cells densely arranged in bundles or vortices (HE×40). **(B)** Elastic fibers are visible (HE×40). **(C)** The boundary between the tumor cells and surrounding striated muscles is unclear, indicating infiltrative growth (HE×40). **(D-H)** IHC results were taken from the left neck lesion: β - Catenin(plasma) was positive **(D)**, Desmin (partial) was positive **(E)**, SMA (partial) was positive **(F)**, CD34 (vascular) was positive **(G)**, and Ki-67 proliferation index was 1% **(H)**.

**Figure 6:**
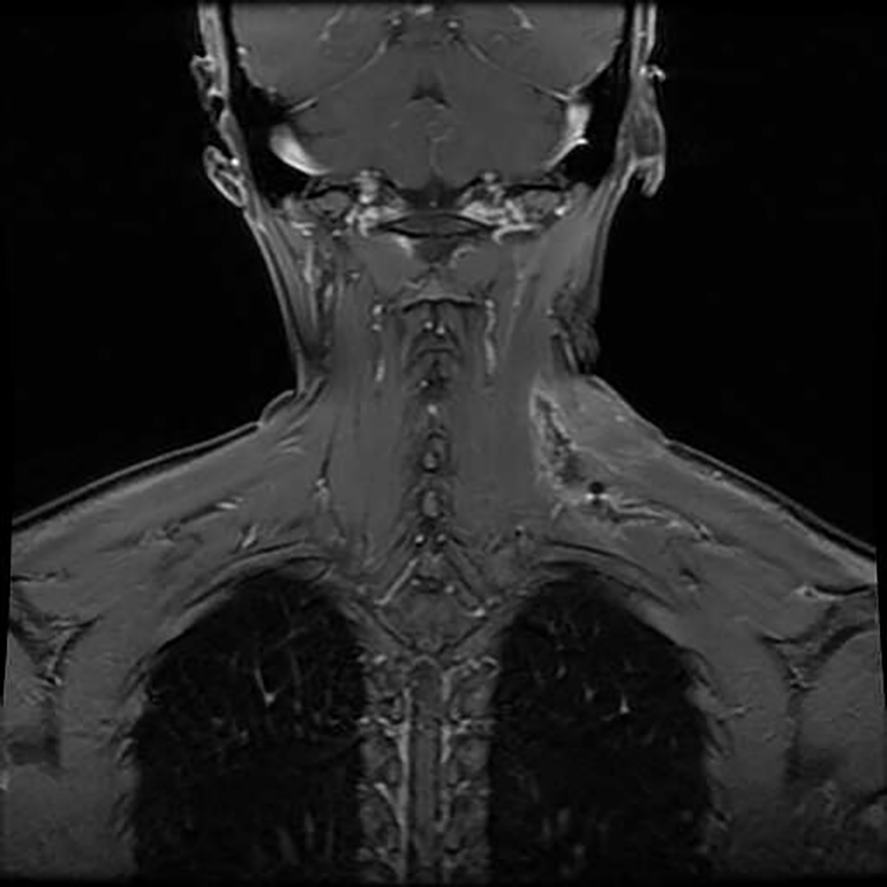
Enhanced MRI images on August 20, 2023: Postoperative changes in the left neck, showing edema and gas accumulation in the surgical area and surrounding soft tissues.

**Figure 7:**
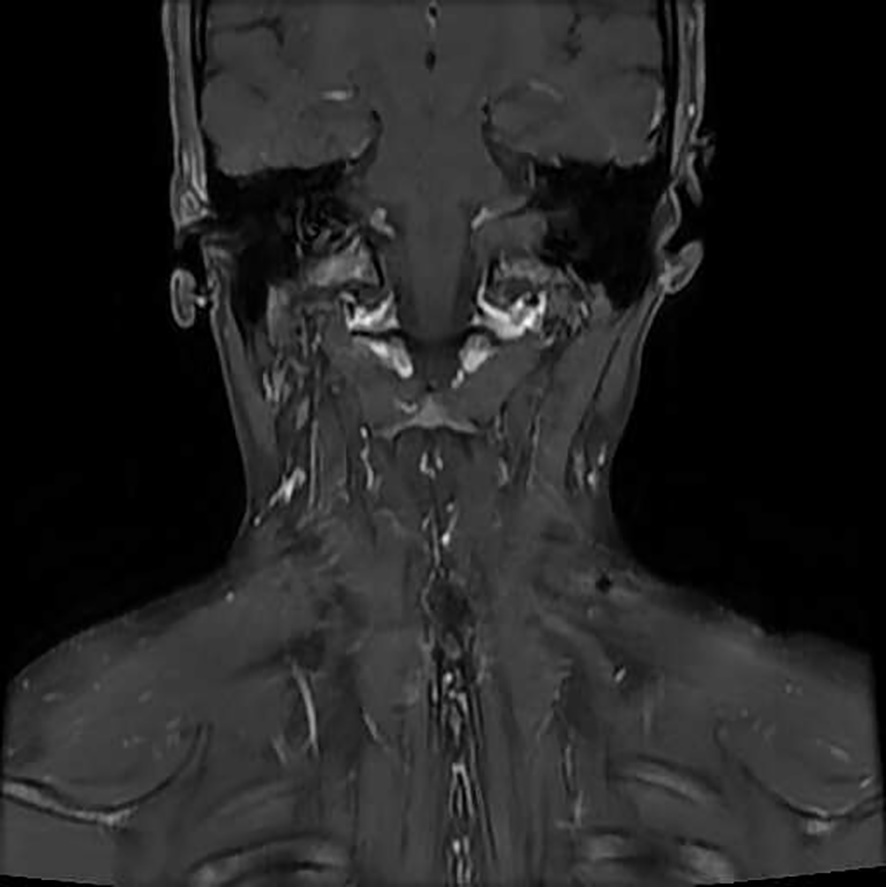
Enhanced MRI images on August 10, 2024: Postoperative changes in the left neck, with local skin depression and multiple small lymph nodes visible in both sides of the neck. No recurrence of the left neck mass was detected during the 12-month follow-up after surgery.

Microscopically, the left neck mass measured 3×7×10 cm, with a grayish-white, moderately tough cross-section. The spindle-shaped tumor cells were arranged in bundles and vortices, and elastic fibers were present (**Figures 5A, B**). The tumor exhibited an infiltrative growth pattern with unclear boundaries with the surrounding striated muscles (**Figure 5C**). Immunohistochemical results showed positive staining for β-catenin (plasma) (**Figure 5D**), partial positivity for Desmin (**Figure 5E**), partial positivity for SMA (**Figure 5F**), and positivity for CD34 (vascular) (**Figure 5G**). Negative results were observed for ALK, S-100, EMA, STAT6, and CDK4. Additionally, the Ki-67 proliferation index was 1% (**Figure 5H**). The pathological diagnosis confirmed aggressive fibromatosis (left neck mass). Frozen section analysis suggested a proliferative lesion of fibroblastic/myofibroblastic cells, consistent with fibromatosis. Postoperatively, the left neck mass was no longer present. On August 20, 2023, an MRI-enhanced scan of the neck soft tissue, performed 5 days after surgery, showed postoperative changes in the left neck, with edema and gas accumulation in the surgical area and surrounding soft tissues (**Figure 6**). On August 23, 2023, the doctor assessed the patient’s recovery as satisfactory and recommended discharge. The patient was advised to return to the neurosurgery clinic 3-4 days after discharge for stitch removal. Nine months later, during a telephone follow-up, the patient reported no discomfort. On August 10, 2024, the patient returned for a follow-up examination and reported no significant discomfort. There was no recurrence found when follow-up was conducted until 12 months post of the operation (**Figure 7**).

## Discussion

Cervical aggressive fibromatosis (AF) is classified as a fibroblastic and myofibroblastic tumor in the WHO (2020) classification of soft tissue tumors (5). AF is extremely rare, representing only 0.03% of all human tumors (6, 7), with an incidence of 2-4 new cases per million people annually. Most cases occur within the abdominal cavity, with only 7%-25% involving the head and neck region. Of those, 40%-85% of fibrous tissue lesions in the head and neck affect the neck (8). According to reports, AF accounts for less than 3% of all soft tissue tumors (9–11), and the incidence of head and neck aggressive fibromatosis is even lower, at less than 6%-8% of systemic AF cases. AF is primarily characterized by a high propensity for local recurrence, with almost no reports of distant metastasis. The prognosis for cervical AF is particularly poor, with a local recurrence rate of 36.8%, significantly higher than the 15.7% recurrence rate of non-head and neck AF (5). Cervical AF was first described as a tumor of the sternocleidomastoid muscle in children (12). Avinçsal et al. reported a large soft tissue mass in the left posterior neck muscles, involving the anterior and middle scalene muscles (13). Chebil et al. described swelling initially limited to the left submandibular region, which gradually extended to the anterior cervical and sublingual areas (14). However, the onset site in this case, within the anterior inferior muscle space of the left dorsal trapezius muscle, is relatively uncommon. When AF occurs in the head and neck region, its invasive growth characteristics may lead to the involvement of surrounding tissues, and it has a tendency to approach critical anatomical structures, increasing the risk of local recurrence. AF is a rare benign fibroblastic lesion. Histologically, these lesions consist of spindle-shaped cells with mild nuclear expression, without evidence of abnormal mitosis or necrosis. Although AF does not metastasize, its tendency for local invasion and recurrence contributes to a significant disease burden, making it closely associated with both morbidity and mortality (15).

AF is more prevalent among women and can occur across all age groups, with peak incidences observed between the ages of 20-40 and 60-69 years (16). In this report, we present the case of a 30-year-old female with lesions originating from the space between the anterior and lower edges of the trapezius muscle in the back of the neck. AF arises from structures such as muscles, fascia, and aponeurosis, exhibiting biological behavior and histopathological characteristics that lie between benign fibrotic lesions and fibrosarcoma. Research suggests that the etiology of this disease may involve genetic factors, environmental influences, trauma, or hormonal changes (17). AF of the neck has shown familial clustering, suggesting a significant role of genetic factors in its pathogenesis. Genetic studies indicate that AF may be linked to specific genetic abnormalities, particularly mutations in the β-catenin gene, which disrupt the normal function of the Wnt/APC/β-catenin signaling pathway, leading to tumor formation. These abnormalities include the inactivation of tumor suppressor genes on chromosomes 8, 20, and 5, as well as mutations in CTNNB1, which encodes β-catenin in adenomatous polyposis of the colon (APC) (18). Previous studies have demonstrated significant β-catenin protein expression in invasive fibromatosis through IHC evaluation, with an overall positive rate ranging from 82% to 100% (19, 20). In this case, IHC analysis revealed nuclear expression of β-catenin protein, consistent with prior literature.

AF manifestations in the head and neck vary widely, ranging from painless masses to painful growths, posing significant diagnostic challenges and potentially delaying treatment. As such, any abnormal lump in the head or neck should undergo comprehensive medical evaluation. While some reports suggest that early symptoms may be mild, over time, the invasive nature of AF can lead to serious complications, such as airway compression and facial deformities (14, 21). During the surgical process, if the tumor is closely connected to important surrounding structures such as nerves and blood vessels, the risk of postoperative complications will increase. Retrospective data of fibromatosis patients treated showed a rate of radiation related complications of 17% (22).

MRI plays a crucial role in the diagnosis of AF (23). It provides a clear visualization of the lesion’s size, location, and its relationship with surrounding tissues, which is vital for surgical planning. Additionally, the low signal intensities observed on T1-weighted and T2-weighted MRI are unique features of collagen fibers in AF (24), contributing significantly to its diagnosis. In the case we report, the initial ultrasound revealed a hypoechoic mass in the lower trapezius muscle of the left neck, with a small amount of punctate blood flow and a clear boundary. The following day, an MRI plain scan showed a mixed signal lesion in the left cervical dorsal muscle space, with equal signal on T1 sequences and a mixed high signal on T2 sequences. The low signal band within the lesion suggested the presence of collagen fibers, and the lesion boundary was well-defined. The initial diagnosis was schwannoma. After 16 months of follow-up, the lesion had slowly enlarged, showing significant improvement on MRI. Low-signal, non-enhanced cord-like areas remained visible within the lesion, indicating low cellularity with collagen fiber composition, which is an important imaging feature supporting the diagnosis of fibromatosis (17). When distinguishing this case from cervical schwannoma, comprehensive evaluation of the patient’s condition is necessary. Schwannomas typically present as soft tissue masses with clear boundaries and a smooth surface, often accompanied by neurological symptoms. On T1-weighted imaging (T1WI), schwannomas exhibit low signal intensity, and MRI enhancement may show uniform or uneven enhancement, though the degree of enhancement is usually lower than that of surrounding muscle. Additionally, schwannomas frequently exhibit internal structural diversity, such as cystic changes or necrotic areas, which appear as low signal intensity on MRI. Fibrous tissue in schwannomas may also result in low signal areas. In contrast, cervical fibromatosis often shows signal characteristics similar to muscle on T1-weighted imaging, with significant enhancement following MRI contrast administration. This distinction, along with other clinical and imaging features, is critical in differentiating fibromatosis from schwannoma. Although there are subtle differences between cervical fibromatosis and cervical schwannoma on MRI, a comprehensive diagnosis must still consider the patient’s medical history, clinical presentation, and other laboratory findings. In this case, the final diagnosis of cervical fibromatosis was confirmed based on pathological and immunohistochemical results.

According to the literature, when the margins of aggressive fibromatosis are clear, extensive surgical resection is the most common first-line treatment (25). Tang et al. also noted that surgical resection is the gold standard for treating extra-abdominal fibroadenoma (26). However, if the tumor is incompletely removed or recurs during follow-up, multimodal therapies such as radiation therapy, hormone therapy, or cytotoxic chemotherapy may be employed as primary or adjuvant treatments (8). It is important to consider preoperative neoadjuvant therapy or radiotherapy and chemotherapy for fibromatosis cases where surgical resection is not feasible due to severe adhesions or multiple tumors (27). In the case of head and neck fibromatosis, particularly in pediatric patients, chemotherapy is often recommended before surgery (28). Commonly used chemotherapy regimens include combinations of methotrexate, vinblastine, doxorubicin, and drugs such as dacarbazine, aimed at disrupting tumor cell growth and division (29). Reports also suggest that tamoxifen, tamoxifen analogs, and medroxyprogesterone acetate may be used as neoadjuvant therapies or as postoperative adjuvant treatments for fibromatosis (30). In the case presented here, the patient was treated with surgical resection of the cervical soft tissue lesion, and did not receive radiotherapy or chemotherapy. The prognosis of AF varies among individuals, but the recurrence rate is generally high. Fortunately, AF does not metastasize to distant sites. Most recurrences in the head and neck region occur within the first two years after surgery but can also happen at any time, from a few months to over ten years postoperatively (8). Therefore, close follow-up and regular monitoring are essential for the timely detection of recurrence and for implementing appropriate treatment measures. In this case, the patient recovered well after surgery without any complications, with no tumor recurrence during the 12-month follow-up, resulting in a favorable prognosis.

The advantage of this report lies in its discovery that AF of the neck originated from a mass located in the anterior inferior muscle space of the left trapezius muscle, a location that has rarely been reported in the literature. This case significantly expands our understanding of the clinical manifestations of AF. Through detailed imaging follow-up and successful surgical treatment, it provides valuable insights into the clinical diagnosis and management of cervical AF. However, it is important to note that this report also has the limitation of a short follow-up period. Therefore, more samples and a period of careful follow-up are needed to evaluate tumor behavior.

## Conclusion

Although simple imaging examination is difficult to accurately diagnose cervical AF, the low signal linear non enhanced areas representing collagen fiber features displayed after enhanced scanning are a key clue that should not be ignored. This case reports a rare case of AF in the anterior inferior margin muscle space of the neck trapezius muscle in an adult female. The patient recovered well after surgical treatment. However, future studies should further expand the sample size and extend the follow-up time to more comprehensively evaluate the imaging characteristics and prognosis of cervical AF, providing a more reliable basis for clinical diagnosis and treatment.

## Data Availability

The original contributions presented in the study are included in the article/supplementary material. Further inquiries can be directed to the corresponding authors.
